# World Rabies Day campaign in the Philippines

**DOI:** 10.1186/s40794-016-0036-7

**Published:** 2016-09-28

**Authors:** Danellie Joy O. Medina, Sarah I. Jayme, Anna Charinna B. Amparo, Rubina O. Cresencio, Emelinda L. Lopez, Mario S. Baquilod, Leda M. Hernandez, Ernesto E. S. Villalon, Louis D. Nel

**Affiliations:** 1 Global Alliance for Rabies Control, Unit 313, 3rd Floor, Humana Building, Balibago-Tagaytay Road, Sta. Rosa City, Laguna Philippines; 2 Department of Agriculture—Bureau of Animal Industry, Visayas Avenue, Quezon City, Metro Manila Philippines; 3 Department of Health—Disease Prevention and Control Bureau, San Lazaro Compound, Tayuman, Sta. Cruz, Manila, Philippines

**Keywords:** Rabies, World Rabies Day, World Health Days, Health communication, Health promotions

## Abstract

**Background:**

Rabies is a fatal disease, claiming the lives of around 59,000 people annually worldwide. It is considered a neglected and underreported disease leading to inadequate support from governments. Apart from dog vaccination and proper animal bite management, an integral part of a successful rabies control program is community education. The Philippine government conducts an extensive nationwide annual World Rabies Day (WRD) celebration as part of its community education.

**Methods:**

Strong inter-sectoral collaboration at the national level is a key factor for the success of WRD, capitalizing on the partners’ strengths to mobilize various sectors. Strategies include the National WRD Celebration and releasing national government memorandums. An invitation letter campaign was initiated, encouraging stakeholders to register their activities. Banners were given as an incentive for those who registered. Mass and social media were also utilized to promote WRD.

**Results:**

Registered WRD events held in the Philippines rose from 10 events in 2012, to 37 events in 2013, to 66 events in 2014 and 76 events in 2015. The individual activities involved veterinary services and information, communication, and education (IEC) activities. Nine unique WRD IEC activities are highlighted in this paper. Promotion of WRD through social media was also utilized in recent years. More news items were published online than those printed in newspapers and aired on television.

**Conclusion:**

The campaign’s success underlines the value of a national government-led program. The national rabies program sets the agenda for priority activities including the WRD campaign. Its capacity to allocate funds for the program also denotes stability which is beneficial for local program implementers. Different segments of society were tapped through various strategies. The campaign’s flexibility allowed for a large range of activities and presented opportunities for expanding partnerships and integration with others interventions for its sustainability. With appropriate tools and government support, the extensive WRD campaign in the Philippines can be replicated in other countries. The strategies discussed prove that since different localities celebrate WRD in their own way, other countries can also organize activities adapted to their culture and contribute to the global campaign against rabies.

## Background

Canine-mediated rabies is a fatal viral disease that continues to kill approximately 59,000 people annually on a global scale [1]. It is mainly transmitted to humans through contact with the saliva, bite, or scratch of an infected animal, most commonly from domestic dogs. Though entirely preventable, death is certain once a person or animal exhibits clinical symptoms.

The World Health Organization (WHO) categorizes rabies among the ‘neglected zoonotic diseases.’ This refers to a group of diseases that are insufficiently addressed by governments and the international community [2]. In most Southeast Asian countries, rabies is still considered a neglected disease due to the diverse priorities and activities of the public health and veterinary services sectors. This neglect influences the perception of policy-makers, leading them to believe that rabies is not of importance. This translates to little or no support for the implementation of disease control measures in their localities [3].

Further proof of the neglected disease status of rabies is that many developing countries still lack the proper facilities and personnel to conduct rabies surveillance and diagnosis [1]. This leads to a lack of reliable data on the actual incidence of deaths due to canine-transmitted rabies since not all cases are notified or reported in these areas [4]. In addition to inadequate resources and limited access to health services, people in rabies-endemic countries usually have a low level of knowledge and awareness of the disease along with its impact [3].

Tragically, it is the communities living in the poorest regions in developing countries that suffer the brunt of rabies. It remains endemic in most parts of Asia and Africa where over 90 % of human rabies deaths occur at present. In rabies-endemic Southeast Asian countries (Cambodia, Indonesia, Lao PDR, Myanmar, Philippines, Thailand, and Vietnam), about 608 million people are at risk of rabies infection and from 2009 to 2013, rabies was reported to have been the cause of death of approximately 3475 people and 7617 animals [3, 5]. Through establishing collaborative public health and veterinary programs, providing accessible health care services, and increasing the awareness of communities about rabies and animal bite prevention and management, lives can be saved and global elimination of the disease can be achieved [3].

In the Philippines, rabies remains a public health concern and is the most fatal infectious disease. At least one-third of these deaths occur in children aged 15 years old and below [6]. Data show that the number of animal bite cases reported in the country increased by 175.69 %, from 2009 (206,253 bite cases) to 2013 (568,629 bite cases). Conversely, the confirmed number of positive human rabies cases decreased by 23.05 % in the last 5 years, from the 243 cases reported in 2009 to 187 in 2013 [5]. Though the number of human deaths has declined over the years, much work still needs to be done as there are existing tools and interventions that make it possible to eliminate canine rabies.

The World Rabies Day (WRD) campaign was created by the Global Alliance for Rabies Control (GARC) in 2007 in partnership with WHO, World Organisation for Animal Health (OIE), United Nations Food and Agriculture Organization (FAO), the Centers for Disease Control and Prevention (CDC), and the Pan-American Health Organization (PAHO) as a response to the call to raise global awareness and mobilize resources for human rabies prevention and animal rabies control. It is celebrated annually every September 28 and is the largest unifying initiative focused specifically on the prevention of human and animal rabies [2].

The goal of the WRD campaign is to increase and sustain global awareness and encourage different sectors to get involved and organize activities that would cascade life-saving information on rabies, animal bite prevention and management, and responsible pet ownership to communities. It also provides a platform to urge governments to support the implementation of national rabies prevention programs [7, 8]. WRD events have been conducted in 150 countries and paved the way for educating 182 million on the disease and vaccinating 7.7 million dogs since its launching in 2007 [2].

GARC serves as the overall coordinator of the campaign including the monitoring and promotion of WRD activities as well as collaborating with partner organizations and governments to extend the campaign’s reach [9]. The Southeast Asian countries have also expressed their full support for the campaign with the inclusion of the WRD celebration under the Socio-Cultural pillar of the Association of Southeast Asian Nations Rabies Elimination Strategy (ARES) [3].

The WRD celebration in the Philippines is anchored on Republic Act No. 9482 also called the Anti-Rabies Act of 2007 [10]. This act mandated the creation of a National Rabies Prevention and Control Program (NRPCP), an inter-sectoral initiative that aims to strengthen the country’s national rabies prevention and control program. The National Rabies Prevention and Control Committee (NRPCC) serves as its implementing body and is composed of representatives from the Department of Health (DOH), Department of Agriculture-Bureau of Animal Industry (DA-BAI), Department of Interior and Local Government (DILG), Department of Education (DepEd), Department of Environment and Natural Resources (DENR), Provincial, City, and Municipal Veterinarians League of the Philippines (PCMVLP), local government units (LGUs), non-government organizations (NGOs), People’s Organizations (PO), and academics [6]. Although such a committee was initiated in 1991, early efforts to eliminate rabies were unsuccessful mainly due to inadequate funding since no definite budget was allotted for the execution of rabies prevention and control activities at the national and local levels [11].

It is the goal of the NRPCP to eliminate rabies and declare the Philippines as a rabies-free country by the year 2020. To achieve this, the Program implements the following key components based on its Manual of Operations—Post Exposure Prophylaxis and Pre-Exposure Prophylaxis; health promotion; dog vaccination; dog population management, a central database system; and responsible pet ownership [6].

Among the strategies adopted by the NRPCP under its health promotion component is the annual WRD celebration in the country. This case study will discuss the strategies of the extensive WRD campaign in the Philippines as a component of its national rabies prevention and control program. It will include data from 2012 to 2015 as documented by GARC.

## Methods

### Inter-sectoral collaboration

**Fig. 1:**
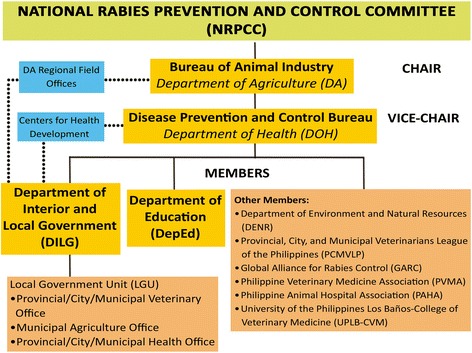
Organizational Structure of the NRPCC

The Philippines, through its national and local government units, has celebrated WRD since its launch in 2007. A crucial factor in the effective implementation of the WRD campaign is the strong collaboration among the various sectors involved. Figure 1 illustrates the NRPCC’s organizational structure [12].

The NRPCC holds several meetings months in advance to prepare for WRD. Information regarding the celebration is then cascaded to the sub-national offices of government committee members. In the case of DILG, information is disseminated to the LGUs from the provincial to the municipal level. This has proven to be effective in encouraging participation of the LGUs as the Philippines implements a decentralized government. This implies that LGUs have the capacity to allocate resources for their rabies control programs including the celebration of WRD in their respective localities through their veterinary and health offices [13]. Other members of the NRPCC include the University of the Philippines Los Baños-College of Veterinary Medicine (UPLB-CVM) representing academics, Philippine Veterinary Medicine Association (PVMA), Philippine Animal Hospital Association (PAHA), and PCMVLP, a national organization comprised of LGU veterinarians. GARC became an official member of the NRPCC in 2014.

As a multi-sectoral committee, the NRPCC espouses a ‘One Health’ approach in controlling and eliminating rabies in the country. The concept of One Health is not new and has been constantly explored and re-discovered over the course of history. While there is no universally accepted definition of One Health, it is fundamentally rooted in the belief that the well-being of humans, animals, and the environment are all interdependent [14].

The One Health approach has gradually gained momentum over the past decades. As the world continuously strives for convergence and globalization, it also brings upon threats and issues that shape the landscape of human health, animal health, and ecosystem health [14]. One Health fittingly addresses these concerns through a holistic and collaborative approach, emphasizing the need for interdisciplinary and inter-sectoral efforts to resolve public health problems [15].

In the context of neglected zoonotic diseases such as rabies, the need for a One Health approach is crucial for disease control and prevention as it entails the collaboration of various stakeholders. While they may use different approaches to control and prevent the disease, these efforts are effectively integrated through the One Health approach [16]. The WRD campaign in the country models how One Health was effectively applied by capitalizing the strengths of its member-agencies and enabling them to mobilize different segments of society to participate in the campaign, from students to policy-makers.

### Strategies for the promotion of WRD

A key feature of the WRD campaign is its flexibility. Provided with a global theme, participants have the opportunity to organize a diverse range of WRD activities that are tailor-made for their country. Fundamental messages on rabies prevention and control can be translated into activities based on their cultural relevance [9]. For the purpose of this review, WRD event organizers per locality will also be referred to as ‘events’ since they have been recorded in the GARC database as a single WRD event. However, LGUs and agencies usually hold multiple individual activities as part of their WRD celebration. These individual WRD activities will be referred to as ‘activities’.

The highlight of the National WRD Celebration is the Declaration of Rabies-Free Zones. Of the seven rabies-endemic countries in Southeast Asia, the Philippines is among only three (along with Indonesia and Thailand) that have an official internal process for declaring rabies freedom in parts of the country [5]. This initiative is jointly handled by the DOH and DA-BAI as mandated by the Joint DOH-DA Order No. 1, Series of 2008 (“Guidelines for Declaring Rabies-Free Zones”) [5]. There are 35 island provinces, municipalities, and villages that have been declared as rabies-free zones to date.

For DA-BAI, WRD is celebrated concurrently with the Animal Welfare Week. This joint celebration highlights the importance of promoting animal welfare as part of rabies elimination. It also seeks to increase public awareness of the importance of vaccinating dogs against rabies [17].

NRPCC member-agencies such as DepEd and DILG have also been equally supportive of the WRD celebration. In 2008, DepEd released a Memorandum Circular enjoining all DepEd units and offices as well as public and private elementary and secondary schools to organize educational and social activities to strengthen rabies prevention education and disseminate information on responsible ownership, immunization, and vaccination of pets [18]. A similar document was released by DILG in 2015 encouraging all local chief executives in provinces, cities, and municipalities to mobilize their respective Provincial and City Veterinary Offices and Municipal Agriculture Offices to conduct and register their WRD activities [19]. These Memorandum Circulars were an important part of the campaign since it was distributed to schools and LGUs nationwide.

Another strategy which was valuable in promoting the WRD celebration was the invitation letter campaign initiated by GARC in close coordination with the NRPCC. Letters were sent to the DOH and DA-BAI sub-national offices, veterinary schools, LGUs, and other government agencies nationwide encouraging them to conduct and register their WRD activities on the GARC website. The letter campaign has evolved through the years, adapting to the advances in technology. From sending letters only through fax in 2012 and 2013, invitations were instead sent to event organizers mainly through email in 2014 and 2015. Online registration of events makes it easy for organizers to add their events to the GARC website themselves. Promoting WRD using social media, particularly through Facebook, was especially helpful in getting the public involved. A “text brigade” or the mass sending of short message service (SMS) or text messages was also initiated by PCMVLP for its members.

As support for local WRD activities, banner tarpaulins (designed in consultation with the NRPCC) were distributed as an incentive for those who registered their activities on the GARC WRD web platform. In 2015, the DOH National Center for Health Promotion led the creation of a standard banner design. Event feedback was obtained by contacting event organizers to thank them for their participation in WRD. A brief update of the activity was also requested along with consent to post of their photos on GARC’s social media accounts. This mechanism allowed GARC to document the outcomes of the activities including the estimated number of people reached by these initiatives.

The WRD celebration was also publicized through mass media. Numerous media outlets nationwide covered the WRD activities held all over the country. News items, press releases, online posts, and video clips, among others released before and after WRD, were monitored and collated.

## Results

### Number of registered WRD events in the Philippines

**Fig. 2:**
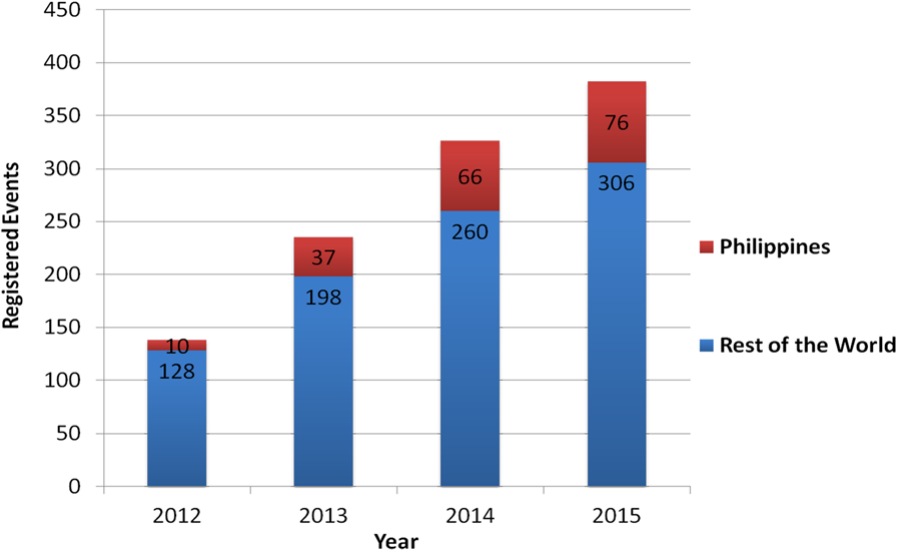
Number of Registered WRD Events in the Philippines and the rest of the world from 2012 to 2015

For the past 4 years (2012 to 2015), the Philippines has registered one of the largest number of WRD events globally, as documented through the GARC WRD platform. ‘Events’ generally refer to a set of activities conducted by a WRD event organizer (e.g. Provincial Veterinary Office, veterinary school, NGO) and organizers may carry out several individual ‘activities’. Figure 2 summarizes the number of registered WRD events held in the Philippines and the rest of the world from 2012 to 2015.

Events held in the Philippines accounted for 8 % of the global total in 2012, rising to 25 % in 2014 and 2015 as seen in Fig. 2. Only two other countries have registered more events between 2012 and 2015 than the Philippines: India and The United States.

**Fig. 3:**
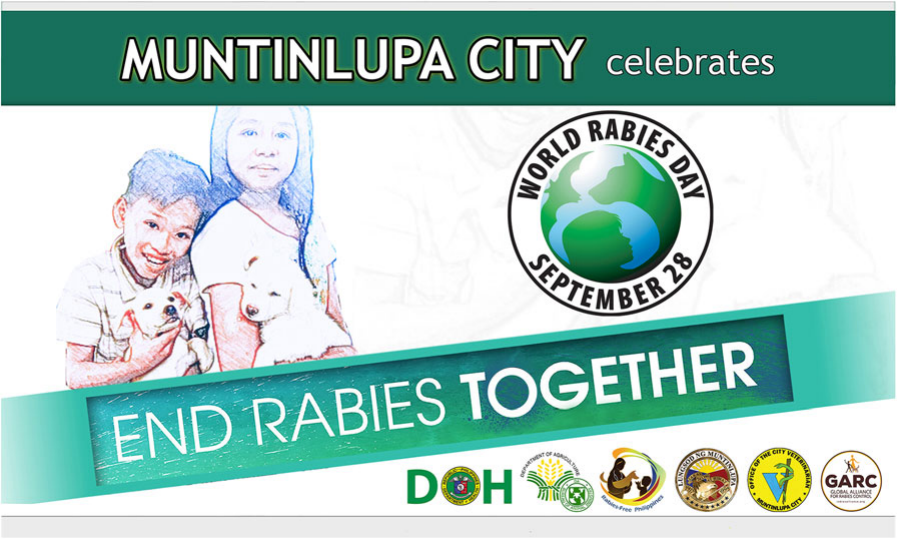
Example of the standard WRD banner tarpaulin distributed to LGUs and other local organizations, agencies, and universities (photo credit: Global Alliance for Rabies Control)

It should be noted though that not all those who were listed were given WRD banners (see Fig. 3). The organizers were given a deadline to register their activities since it would take a minimum of 2 weeks to print and send the banners via courier. For those that did not meet the deadline, their activities were gathered based on the updates on the official Facebook pages and websites of the LGUs and agencies. Table 1 shows the summary of registered WRD events in the Philippines.

**Table 1:** Summary of registered WRD contributors/events in the Philippines from 2012 to 2015

Year	Breakdown of events						Total number of registered events
	Local government units	DA-regional field offices	DOH regional offices	National government agencies	Universities/veterinary schools	Private organizations	
2012	6	–	–	–	–	4	10
2013	37	–	–	–	–	–	37
2014	55	1	–	3	2	5	66
2015	61	4	1	4	4	2	76

As the letter campaign was aimed at the LGUs, most of the event organizers, not surprisingly came from this sector. For 2015, the large number of LGUs that registered can also be attributed to the Memorandum Circular from DILG that were sent out. In some cases, the sub-national offices assisted the LGUs in their WRD activities.

### Examples of individual WRD activities

As part of the national WRD celebration, local governments throughout the country conducted activities such as mass dog vaccination and information, education, and communication (IEC) campaigns to coincide with the launch of WRD activities each year. While there is a national directive for LGUs and agencies to conduct activities in celebration of WRD, only a portion of the estimated total number of these activities was documented through the invitation letter campaign. Table 2 shows the summary of individual WRD activities conducted in the Philippines. The activities were grouped into two major categories: a) veterinary services and b) IEC campaign.

**Table 2:** Summary of individual WRD activities conducted in the Philippines

WRD activity	Examples of individual WRD activities	Year			
		2012	2013	2014	2015
A. Veterinary Services	- Mass dog vaccination - Free pet health consultation - Deworming - Spay and neuter	10	42	82	72
B. IEC campaign	The total number of IEC activities were further grouped into three sub-categories: 1. Community and School-based Activities - Seminar/lecture/forum/symposium on rabies prevention and control and responsible pet ownership - Distribution of educational materials - Film show 2. Community-based Activities - Radio and TV interviews - Media Forum - Rabies Summits - Pet Blessing - Dog Walk/Fun Run - Dog Show - Motorcade - LGU-based competition (e.g. Recognition of Most Pet-Friendly Village and Rabies Program Partners, Awarding of Rabies-free Villages) - Signing of Anti-Rabies Covenant 3. School-based activities - Poster-making contest - Jingle-making contest - Trash-to-art making contest - Rabies Quiz Bee	13	55	98	88
TOTAL		23	97	180	160

While there were more IEC activities conducted over the years (since it covered a wider range of activities), the veterinary services were an equally significant element of the WRD celebration in the Philippines. The greater part of the activities in this category involved mass dog vaccination which was generally accompanied by lectures and seminars educating the community on the impacts of rabies and the importance of having their dogs and cats vaccinated against the disease. LGU veterinarians usually provide these services for free during WRD to promote their rabies control and elimination programs and encourage more people to avail of the services. For the mass dog vaccination, staff from the Provincial and City Veterinary Office or Municipal Agriculture Office either went from house to house or set up vaccination stations in public areas (e.g. village hall or basketball court) to ensure that the majority of the dogs and cats in the locality were vaccinated.

**Fig. 4:**
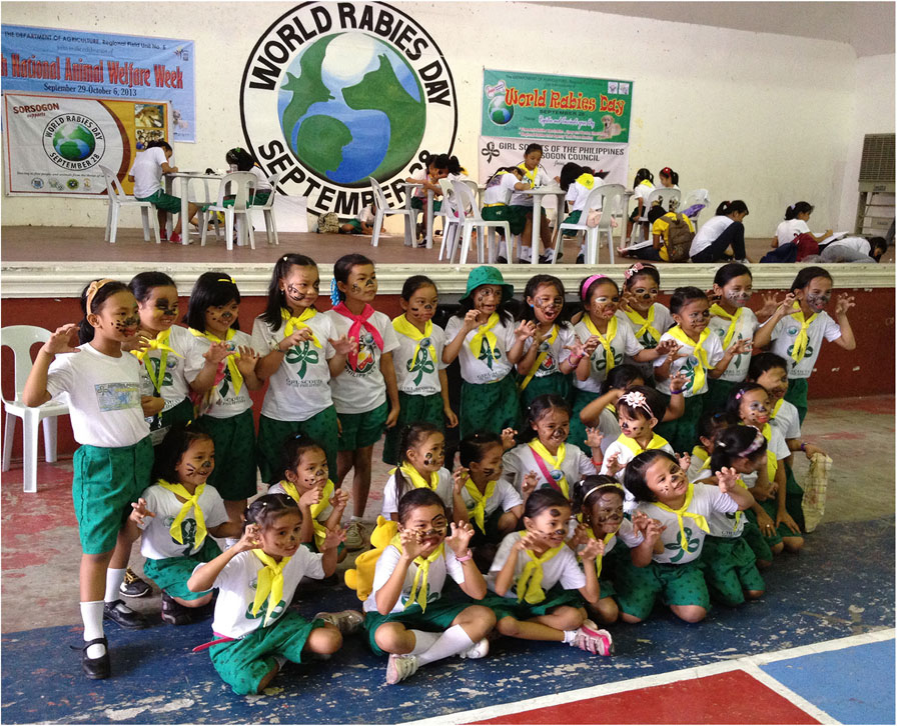
WRD celebration of the Girl Scouts in the province of Sorsogon (photo credit: Global Alliance for Rabies Control)

The organizers have shown great imagination and effort in conducting the IEC campaign with activities ranging from fun runs and dog shows, to various school-based and community-based competitions, and the use of mass media, among others. Based on organizers’ feedback, it is estimated that more than 100,000 Filipinos were reached and educated on rabies prevention and control through the various community and school-based IEC activities held as part of WRD. For the purpose of this paper, we have chosen to highlight nine innovative WRD IEC activities conducted in the Philippines over the years. Table 3 lists examples of these activities and a brief description of each (Fig. 4).

**Table 3:** Examples of innovative WRD IEC activities conducted in the Philippines

Activity	Organizer	Description of activity
School-Based WRD Activities		
1. Trash-to-Art making contest	City Veterinary Office, Marikina City, Metro Manila	With the theme, *“Alaga Ko, Mahal Ko”* (I Love My Pet), high school students participated in the competition which aimed to encourage the use of creative approaches such as the visual and performing arts to intensify the present awareness of the community on rabies.
2. WRD activities in coordination with the Girl Scouts of the Philippines (GSP) Council-Sorsogon	GSP Council; Provincial Veterinary Office, Province of Sorsogon	The GSP Council has been regularly conducting WRD activities that promote key messages on rabies, animal bite prevention and management, and responsible pet ownership among elementary and high school students since 2013 in partnership with the Provincial Veterinary Office (see Fig. 4).
3. Third Provincial Rabies Quiz Bee	Provincial Veterinary Office, Province of Ilocos Norte	Elementary students joined the quiz bee which was held to raise rabies awareness and responsible pet ownership among the students and their parents. The questions were based on the Rabies Education Manual currently being used in the province.
4. PRO DOG: Pet Responsible Ownership of Dogs	Armed Forces of the Philippines Veterinary Dispensary, Quezon City, Metro Manila	Elementary students along with their parents were educated on rabies, responsible pet ownership, dog vaccination, and proper bite management during the activity. The children had a storytelling and arts and crafts session including coloring of dog masks, origami-making and dog puppet - making while the parents attended a lecture.
Community-based WRD Activities		
5. *Baklay* 2013: Third Walk for a Cause	Alrae Veterinary Clinic, Municipality of Maramag, Province of Bukidnon	A Dog Walk was conducted to promote responsible pet ownership among members of the community. Other activities were conducted including a Dog Bite Prevention and Rabies Awareness Seminar and free veterinary services such as anti-rabies vaccination, consultation, and spay and neuter.
6. Search for Rabies-free Village 2015	City Veterinary Office, Muntinlupa City, Metro Manila	With the aim to declare Muntinlupa City rabies-free by the year 2018 and be the first city in the National Capital Region to be declared as such, this activity was conducted to further strengthen collaboration and cooperation between stakeholders and officials at the village level, increase rabies awareness, and promote responsible pet ownership in the city.
7. Second Tayabas Run Against Rabies	City Veterinary Office, Tayabas City, Province of Quezon	An activity that promoted both rabies prevention and health and wellness, the Fun Run was made possible through the participation of various sectors and agencies including teachers, students, police officers, local officials and rabies councils as well as the general public.
WRD Activities for Policy-Makers		
8. One Health Summit on Rabies and Food Animal Diseases	UPLB-CVM in coordination with the Provincial Veterinary Office and Health Office of Laguna	Emphasizing the importance of the One Health approach, the activity served as a venue for different health professionals and local chief executives to discuss ground level implementation of rabies control and elimination programs. The Summit also gave an opportunity for the participants to share community issues, experiences, and best practices.
WRD Activities for Out-of-School Youth		
9. Si Bantay at Ako goes to He Cares Foundation	GARC in coordination with: - He Cares Foundation Street Children Caring Center - Marikina City Veterinary Office - Student Organizations from the University of the Philippines Los Baños	Organized as part of GARC’s Out-of-School Intervention, various activities including a puppet show, group discussion, and arts and crafts session were conducted to educate and engage out-of-school and street children in urban communities specifically on animal bite prevention and responsible pet ownership.

### Promotion of the WRD celebration

Various communication channels were also utilized to further promote the observance of WRD to a wider audience. Table 4 provides a summary of the number of news items that were printed, posted online, and aired on television as part of the WRD celebration.

**Table 4:** Number of newspaper, online, and televisions news items about the WRD celebration

Types of communication channels	Year				TOTAL (number of news items produced per communication channel for each year)
	2012	2013	2014	2015	
1. Newspaper articles/advertisements	7	3	1	–	11
2. Online articles	–	14	16	31	61
3. Television interviews/programs	–	1	1	4	6
TOTAL (number of news items produced for each year)	7	18	18	35	

**Fig. 5:**
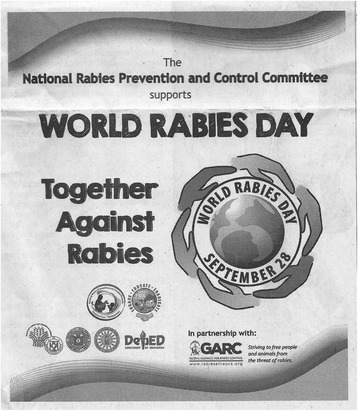
Example of World Rabies Day print advertisement in a national newspaper (photo credit: Global Alliance for Rabies Control)

In 2012 and 2014, print advertisements (see Fig. 5) were placed in some of the most widely-circulated newspapers in the country with millions of estimated daily readers nationwide to promote the WRD celebration. A number of regional and national newspaper outlets likewise covered the WRD activities held in various parts of the country. All in all, over nine million readers were estimated to have been informed of the WRD celebration through print media from 2012 to 2015.

Promotion through radio and television was also essential in involving more people in the campaign. Several major television networks covered the national WRD celebration. Other networks invited members of the NRPCC on to their shows to talk about WRD, rabies, animal bite prevention and control, and responsible pet ownership [20].

While the contributions of print media and television cannot be disregarded, placing an advertisement in the primary segments of newspapers was costly. Hence, social media has been increasingly utilized to promote WRD to the general public over the years since most newspaper outlets and television stations also have online portals and Facebook pages. Filipinos are among the most active internet users in Southeast Asia, spending an average of five hours daily using the internet. There are also 47 million active Facebook users in the country as of 2015 which makes it a more practical and cost-effective platform to promote the campaign and reinforce key messages on rabies control and prevention [21].

In 2014, a digital advertisement was placed on the Philippine Star’s website which ran on its leaderboard on five pages (Homepage, Headlines, Business, Entertainment, and Lifestyle) for 5 days that extended after WRD. The advertisement was linked to GARC’s website to provide more information on the campaign. Online articles about WRD posted by different websites were also monitored and collated. These include articles from newspaper, radio, and television online portals, official websites of LGUs and national government agencies including the Philippine Information Agency, and personal web blogs, to cite a few. The NRPCC promoted individual WRD activities on their official Facebook page as well [22].

## Discussion

Rabies continues to be a largely underreported and neglected disease in many developing countries. The majority of rabies cases remain unreported hence there is a lack of accurate data reflecting the grave impacts of the disease. As a result, rabies remains under the radar of most government policies and programs and this is considered one of the challenges in controlling canine-mediated rabies in rabies-endemic areas [1].

The Philippines is among the eight Southeast Asian countries that have committees designated to specifically address and prioritize the prevention and control of rabies [5]. The success of the WRD campaign in the country demonstrates how valuable it is that a campaign of this scale is led and supported by the national government. The NRPCC’s leadership provides direction for the national rabies prevention and control program and sets the agenda for priority activities which include the WRD campaign, among others. With its capacity to allocate funds specifically for the control and prevention of rabies, its presence also denotes stability for the program. As the Philippines implements a decentralized government, this is extremely beneficial for rabies programs implementers at the LGU level to sustain their activities and seek the support of their local policy-makers.

The NRPCC’s inter-sectoral approach has also been an essential factor in encouraging the participation of the public in the WRD celebration. Each sector is appropriately represented through its members, which allows the NRPCC to maximize the strengths of each agency and its networks. Notable examples are the Memorandum Circulars released by DILG and DepEd to tap local policy-makers and students, respectively to celebrate WRD. It also highlights the importance of a close partnership between the public and private sector since each agency and organization has something to offer to make the campaign a success.

While campaign organizers have already made great strides in the celebration of WRD, there are still opportunities to further improve it. Its annual celebration can open outlets for other interventions and for the integration of other diseases. For instance, the Provincial Veterinary Office of Ilocos Norte plans to include other diseases such as dengue in their annual Provincial Rabies Quiz Bee. On the other hand, the Marikina City Veterinary Office was able to tackle two community issues (i.e. conserving the environment and rabies control and prevention) through their Trash-to-Art contest for students. Inter-sectoral networks can also be expanded to include other partners. Since WRD is celebrated alongside the Animal Welfare Week, different Animal Welfare groups can be involved in the implementation of the campaign, sharing the same advocacy effort.

Based on the results of the invitation letter campaign, which was the main method used to gather information, there is reason to believe that the number of WRD activities that took place in the country far exceed those that were registered. The strategies mentioned in this review each possess strong and weak points. The letter campaign in particular was valuable in promoting WRD to different stakeholders. The LGUs responded positively to the campaign particularly on the provision of banners. To sustain their active participation in the campaign including the registration of their WRD events, a potential way forward would be to capitalize on the strength of social media. With the fast-paced advancements in technology and the growing number of social media users in the country, the possibility of registering WRD activities through Facebook can be explored. For a more thorough analysis of the impacts of the activities, the feedback mechanism can also include interviews and surveys for the organizers.

In addition to WRD, the Philippines also celebrates Rabies Awareness Month annually every March as mandated by a Presidential Executive Order. Given that WRD is a 1-day event, the government can use this month to keep up the momentum in raising the public’s awareness and strengthening rabies control and prevention efforts nationwide.

The activities mentioned here are just a small number of the overall picture of the WRD celebration in the Philippines but we believe that the wide range of activities discussed in this paper more than adequately demonstrates the adaptability of the campaign. With a global theme as a guide, organizers are given the opportunity to think outside the box and create innovative activities for different segments of society. The WRD campaign also recognizes and intensifies the existing rabies control programs and activities of the partners. This is especially true for local rabies program implementers who showcase their regular dog vaccination and IEC activities by providing free services during the WRD celebration. These activities can likewise be replicated by other countries and adapted based on their culture and traditions.

## Conclusion

In countries such as the Philippines where rabies is still endemic, the WRD celebration presents an opportunity to educate and raise the awareness of national and local government staff as well as communities on how fatal rabies is, and call for action for the elimination of this deadly disease.

The World Rabies Day campaign in the Philippines owes its success to the enthusiasm and active involvement of the public and private sectors in the country. It clearly demonstrates that an extensive campaign can be achieved through multi-sectoral support and participation.

The strategies discussed also demonstrate that different localities were able to celebrate WRD in their own unique way. Therefore, other countries can also organize their own WRD activities, no matter how big or small, and contribute to the global campaign against rabies.

## Abbreviations

ARES: Association of Southeast Asian Nations Rabies Elimination Strategy
CDC: Centers for Disease Control and Prevention
DA-BAI: Department of Agriculture-Bureau of Animal Industry
DENR: Department of Environment and Natural Resources
DepEd: Department of Education
DILG: Department of Interior and Local Government
DOH: Department of Health
FAO: United Nations Food and Agriculture Organization
GARC: Global Alliance for Rabies Control
GSP: Girl Scouts of the Philippines
IEC: Information, Education, and Communication
LGU: Local Government Unit
NGO: Non-Government Organization
NRPCC: National Rabies Prevention and Control Committee
NRPCP: National Rabies Prevention and Control Program
OIE: World Organisation for Animal Health
PAHA: Philippine Animal Hospital Association
PAHO: Pan-American Health Organization
PCMVLP: Provincial, City, and Municipal Veterinarians League of the Philippines
PVMA: Philippine Veterinary Medicine Association
SMS: Short Message Service
UPLB-CVM: University of the Philippines Los Baños-College of Veterinary Medicine
WHO: World Health Organization
WRD: World Rabies Day

## References

[1] Hampson K, Coudeville L, Lembo T, Sambo M, Kieffer A, Attlan M, et al. Estimating the Global Burden of Endemic Canine Rabies. PLOS Neglected Tropical Diseases. 2015. http://dx.doi.org/10.1371/journal.pntd.0003709. Accessed 4 July 2016. 10.1371/journal.pntd.0003709 PMC4400070 25881058

[2] WHO Expert Consultation on Rabies: Second Report. WHO Technical Report Series No. 982. 2013. http://apps.who.int/iris/bitstream/10665/85346/1/9789240690943_eng.pdf?ua=1. Accessed 1 July 2016. 24069724

[3] OIE ASEAN Rabies Elimination Strategy. 2015. http://vncdc.gov.vn/files/article_attachment/2015/3/endorsed-ares-final.pdf. Accessed 1 July 2016.

[4] Taylor LH, Hampson K, Fahrion A, Abela-Ridder B, Nel LH. Difficulties in Estimating the Human Burden of Canine Rabies. Acta Tropica. 2015. http://www.sciencedirect.com/science/article/pii/S0001706X15301844. Accessed 29 July 2016. 10.1016/j.actatropica.2015.12.007 PMC5178864 26721555

[5] OIE Benchmark Document: Rabies and Rabies-Related Initiatives in ASEAN Member States. 2014. http://www.rr-asia.oie.int/fileadmin/SRR_Activities/STANDZ/Benchmark_Document_Final_V7.pdf. Accessed 5 July 2016.

[6] National Rabies Prevention and Control Program Manual of Operations. 2012. http://www.doh.gov.ph/sites/default/files/publications/FINALMOP6.4.13WORDRADMay30.pdf. Accessed 28 June 2016.

[7] Costa P, Briggs D, Tumpey A, Dedmon R, Coutts J. World Rabies Day Outreach to Asia: Empowering People Through Education. Asian Biomedicine. 2009. https://rabiesalliance.org/uploads/media/Scientific_resources/World_Rabies_Day/4._WRDABM-AUG-A20093451.pdf. Accessed 20 May 2016.

[8] Rabies Control—Towards Sustainable Prevention at the Source. Compendium of the OIE Global Conference on Rabies Control. 2011. www.oie.int/doc/ged/d12061.pdf. Accessed 18 July 2016.

[9] Nel L, Taylor. Global partnerships are critical to advance the control of Neglected Zoonotic Diseases: The case of the Global Alliance for Rabies Control. Acta Tropica. 2015. https://www.researchgate.net/publication/283444416. Accessed 18 July 2016. 10.1016/j.actatropica.2015.10.014 26519885

[10] Republic Act No. 9482: Anti-Rabies Act of 2007. http://www.gov.ph/2007/05/25/republic-act-no-9482/. Accessed 20 May 2016.

[11] National Rabies Prevention and Control Program Medium-term Plan 2012–2016. http://www.doh.gov.ph/sites/default/files/publications/FINAL_MTP_Rabies.pdf. Accessed 28 June 2016.

[12] WHO Global Elimination of Dog Mediated Human Rabies Global Conference—Presentations. 2015. http://www.oie.int/fr/RABIES2015/presentation/Session_5.2.Cresencia_%20Impact_Recipient_Country.pdf. Accessed 14 July 2016.

[13] Department of Interior and Local Government. The Local Code of the Philippines. http://www.dilg.gov.ph/PDF_File/reports_resources/dilg-reports-resources-2016120_fce005a61a.pdf. Accessed 18 July 2016.

[14] Evans BR, Leighton FA. A History of One Health. Revue Scientifique et Technique de l’OIE. 2014. http://www.oie.int/doc/ged/D14076.PDF. Accessed 9 Sept 2016. 10.20506/rst.33.2.2298 25707172

[15] Papadopoulos A, Wilmer S. One Health: A Primer. National Collaorating Centre for Environmental Health. 2011. http://www.ncceh.ca/sites/default/files/One_Health_Primer_Nov_2011.pdf. Accessed 9 Sept 2016.

[16] Fourth International Meeting Control of Neglected Zoonotic Diseases: From Advocacy to Action. World Health Organization. 2014. http://apps.who.int/iris/bitstream/10665/183458/1/9789241508568_eng.pdf?ua=1. Accessed 10 Sept 2016.

[17] DA-BAI to Hold World Rabies Day and Animal Welfare Kick-Off Celebration. https://www.facebook.com/AgriPinoy/photos/a.524349634313148.1073741831.521426187938826/702434749837968/?type=3&theater. Accessed 2 July 2016.

[18] Department of Education Memorandum Circular No. 438 Series of 2008. http://www.deped.gov.ph/sites/default/files/memo/2008/DM_s2008_438.pdf. Accessed 28 June 2016.

[19] Department of Interior and Local Government Memorandum Circular No. 2015–105 Series of 2015. http://www.dilg.gov.ph/issuances/mc/2015-World-Rabies-Day-Celebration-on-the-28th-Day-of-Septemberin-Conjunction-with-the-Animal-Welfare-Week-Celebration/2194. Accessed 28 June 2016.

[20] MedTalk Episode 97: Rabies. https://www.youtube.com/watch?v=cMexUy2OxBc. Accessed 7 July 2016.

[21] Digital, Social, and Mobile in APAC 2015. We Are Social and IAB Singapore’s Compendium of Asia-Pacific Digital Statistics. http://wearesocial.com/sg/special-reports/digital-social-mobile-in-apac-in-2015. 2015. Accessed 18 July 2016.

[22] National Rabies Prevention and Control Committee facebook page. https://www.facebook.com/groups/771234572943301/. Accessed 7 July 2016.

